# The performance of community health workers in the management of multiple childhood infectious diseases in Lira, northern Uganda – a mixed methods cross-sectional study

**DOI:** 10.3402/gha.v9.33194

**Published:** 2016-11-22

**Authors:** Phillip Wanduru, Moses Tetui, Doreen Tuhebwe, Michael Ediau, Monica Okuga, Christine Nalwadda, Elizabeth Ekirapa-Kiracho, Peter Waiswa, Elizeus Rutebemberwa

**Affiliations:** 1 Makerere University School of Public Health (MakSPH), Makerere University, Kampala, Uganda; 2 Umeå International School of Public Health (UISPH), Umeå University, Umeå, Sweden; 3 Global Health Division, Karolinska Institutet, Solna, Sweden

**Keywords:** community health workers, childhood, infectious diseases, mortality, performance, malaria, diarrhea, pneumonia, rural

## Abstract

**Background:**

Community health workers (CHWs) have the potential to reduce child mortality by improving access to care, especially in remote areas. Uganda has one of the highest child mortality rates globally. Moreover, rural areas bear the highest proportion of this burden. The optimal performance of CHWs is critical. In this study, we assess the performance of CHWs in managing malaria, pneumonia, and diarrhea in the rural district of Lira, in northern Uganda.

**Designs:**

A cross-sectional mixed methods study was undertaken to investigate the performance of 393 eligible CHWs in the Lira district of Uganda. Case scenarios were conducted with a medical officer observing CHWs in their management of children suspected of having malaria, pneumonia, or diarrhea. Performance data were collected using a pretested questionnaire with a checklist used by the medical officer to score the CHWs. The primary outcome, CHW performance, is defined as the ability to diagnose and treat malaria, diarrhea, and pneumonia appropriately. Participants were described using a three group performance score (good vs. moderate vs. poor). A binary measure of performance (good vs. poor) was used in multivariable logistic regression to show an association between good performance and a range of independent variables. The qualitative component comprised seven key informant interviews with experts who had informed knowledge with regard to the functionality of CHWs in Lira district.

**Results:**

Overall, 347 CHWs (88.3%) had poor scores in managing malaria, diarrhea, and pneumonia, 26 (6.6%) had moderate scores, and 20 (5.1%) had good scores. The factors that were positively associated with performance were secondary-level education (adjusted odds ratio [AOR] 2.72; 95% confidence interval [CI] 1.50–4.92) and meeting with supervisors in the previous month (AOR 2.52; 95% CI 1.12–5.70). Those factors negatively associated with CHW performance included: serving 100–200 households (AOR 0.24; 95% CI 0.12–0.50), serving more than 200 households (AOR 0.22; 95% CI 0.10–0.48), and an initial training duration lasting 2–3 days (AOR 0.13; 95% CI 0.04–0.41). The qualitative findings reinforced the quantitative results by indicating that refresher training, workload, and in-kind incentives were important determinants of performance.

**Conclusions:**

The performance of CHWs in Lira was inadequate. There is a need to consider pre-qualification testing before CHWs are appointed. Providing ongoing support and supervision, and ensuring that CHWs have at least secondary education can be helpful in improving their performance. Health system managers also need to ensure that the CHWs’ workload is moderated as work overload will reduce performance. Finally, although short training programs are beneficial to some degree, they are not sufficient and should be followed up with regular refresher training.

## Introduction

Globally, there has been a major reduction in child mortality rates from 12.9 million deaths in 1990 to 5.9 million deaths in 2015 (1). Wide inequalities still exist in the distribution of the global child mortality. The less developed parts of the world bear most of the burden (2). For instance, almost half of this global burden is contributed by the sub-Saharan region of Africa (3). One in twelve children is likely to die before the age of 5 in sub-Sahara. In contrast, only 1 in 147 children is likely to die before the same age in high-income countries (1).

Uganda, a country in the sub-Sahara region, has had a considerable reduction in child mortality in the recent past; however, it still ranks high among countries with the highest child mortality burdens in the world (4). A staggering 167,000 children below the age of 5 years are lost every year in Uganda (1). Infectious diseases, in particular malaria, pneumonia, and diarrhea, are the major causes of mortality among these children (3). Deaths due to these diseases are largely preventable through simple interventions.

One such intervention that has been shown to considerably reduce these deaths is the use of community health workers (CHWs) to diagnose and treat childhood diseases (5). The CHWs can potentially improve access to basic health services in all parts of the country. Other low-income countries such as Ethiopia and Bangladesh have managed to achieve their Millennium Development Goal targets for child mortality by working with CHWs (6–8).

In an effort to improve child survival, CHWs were introduced into the health care system in Uganda in 2011. The CHWs are volunteers who are selected by their communities and undergo a short period of training. Every village in northern Uganda has two CHWs who are trained to manage childhood malaria, diarrhea, and pneumonia. Their major role is to improve health through increasing access to services even in the most remote of rural villages (9).

A number of studies have been undertaken to investigate the performance of CHWs in managing childhood diseases (10, 11). These studies mainly concluded that CHWs had the knowledge and skills to perform their duties adequately. However, these studies were conducted on CHWs who managed only one or two of these childhood diseases. The CHWs in Uganda now typically manage three childhood illnesses. Moreover, most of the previous studies were conducted as pilots by research institutions and non-government organizations covering smaller areas, and therefore they were relatively easy to implement. However, the findings of pilot projects are not always generalizable to the national level where the breadth of coverage is greater, and implementation and monitoring are more complex.

The Lira district in northern Uganda is predominantly rural and has peculiar characteristics that make it stand out from the rest of the country. This region suffered during the civil war waged against the Ugandan government by the Lord's Resistance Army (LRA) for over 20 years. Many health system structures were destroyed by war and to date there are very few functional health facilities. The child mortality rates are higher at 105/1,000 live births compared with the national average of 90/1,000 live births (12). In addition, human resources in health care are limited (13). The CHWs in northern Uganda, therefore, have a very critical role to play in reviving the health system.

In Lira, CHWs were active during the war even though the program had not been officially adopted by the government. During this time, the majority of the population was still living in internally displaced peoples’ camps, and CHWs were just volunteers helping medical teams but without training. Now that the war has ended, every village has five CHWs, two of whom are trained to manage childhood malaria, diarrhea, and pneumonia. While treating children, they follow the integrated management of childhood illnesses (IMCI) treatment algorithm to identify signs and symptoms to make the final decision on the course of management. The CHWs are able to administer oral rehydration therapy, the first-line treatment for malaria (artemether 20 mg and lumefantrine 120 g) and oral antibiotic treatment (amoxicillin 125 mg). However, the supply of these medicines has not been consistent in the recent past. In addition, the CHWs are expected to refer and follow-up children who need further care.

In this study, we assessed the performance of CHWs in managing malaria, diarrhea, and pneumonia in the Lira district, and we investigated associated predictors. Performance was measured using a combination of knowledge assessment and case management, which has been paramount because it enabled evaluation of both knowledge and skills ensuring better congruence with actual job performance (14, 15). The study findings will be used to inform policy makers about the kind of strategies that can be used to improve the performance of CHWs in the mainstream health system. These will help to increase the number of children accessing good quality healthcare services, and in the long run will reduce child mortality in the district of Lira.

## Designs

### Study setting

The study was conducted in the Lira district, northern Uganda, from June to July 2015. Lira has a population of about 368,100 people (12). It is divided into four counties: Erute north, Erute south, Moroto, and Lira municipal, with each of these being a health sub-district. Below the level of a county, the next administration structure is a sub-county, followed by the parish, and finally by a village. CHWs serve at the level of a village. A village in Uganda is estimated to have 100 households; however, due to continuous demographic changes, this can alter. All CHWs are attached to a nearby low-level facility usually at the parish or sub-county level, and here they are required to provide regular reports about their activities. The five major causes of childhood morbidity are malaria, pneumonia, diarrhea, skin diseases, and intestinal worms (16).

### Study design

This was a cross-sectional mixed methods study. It used quantitative and qualitative approaches to assess CHW performance for the community-based management of malaria, diarrhea, and pneumonia in children in the Lira district of Uganda. The study included all CHWs who were actively managing childhood illnesses in their communities and who consented to take part in the study. We excluded those who had not actively participated in the care of children in the preceding 6 months.

### Sample size and sampling procedure

The required sample size for this study was 428 respondents (17). One sub-county was randomly selected from each of the four counties in Lira to take part in the study: these were the Abako, Adwari, Barr, and Adyel sub-counties.

A list of all CHWs in the selected sub-counties was acquired from the District Health Office. The CHW coordinators reviewed the lists and removed those who were no longer active. Probability proportionate sampling was then used to get sample size for each of the selected sub-counties. After determining the number of respondents required from each of the selected sites, a random selection of respondents was undertaken using a table of random numbers.

Seven key informant interviews (KIIs) were conducted with the District Health Officer (DHO) of Lira, the Assistant District Health Officer (ADHO), the District Health Educator (DHE), and the four CHW coordinators in the district, using a pretested interview guide. The key informants (KIs) were purposively selected because of their expert knowledge with regard to the roles and functions of CHWs in the district.

### Study tools and data collection

A knowledge assessment questionnaire was administered orally by trained data collectors, and it was followed by a case scenario. The questionnaires contained questions assessing signs, symptoms, and prescription of drugs for malaria, pneumonia, and diarrhea. It was adopted from a study on Home-Based Management of Fever (HBMF) (18) and the questions were modified using the CHWs training manuals provided by the Ministry of Health (MoH), Uganda (19).

In the case scenarios, sick children who had come to seek care in health center IIs were purposively selected for the study. Health center IIs are low-level facilities and they often receive moderately sick children. A medical officer with training in IMCI participated in each case scenario. They would identify moderately sick children who could be managed by CHWs, obtain consent from their parents, and observe and score the process of managing the children by CHWs. The checklist was used to score the CHWs. It was designed based on Integrated Community Case Management (ICCM) management algorithms of malaria, diarrhea, and pneumonia. The CHWs were expected to demonstrate an ability to identify the signs and symptoms of these illnesses as well as prescribe proper medications. After the case scenarios, the participating children were subsequently managed by the medical officer and treated appropriately.

A pretested KII guide was administered orally to KIs. The guide included open-ended questions, and all interviews were audio-recorded.

### Measurement of performance

As an outcome variable, CHW performance was defined as the ability of the CHW to identify signs and symptoms, and prescribe medicines appropriately (11). In the study, we assessed performance by combining scores from knowledge assessment and case management.

To describe the performance of CHWs in managing multiple illnesses, scores from the knowledge assessment and case management were converted to percentages and then categorized into good, moderate, and poor scores. A score of 0–49% was classified as poor, 50–74% as moderate, and 75–100 score as good (20). A binary performance variable was used to assess factors associated with (predictors of) CHW performance. The combined scores from the knowledge assessment and case scenarios were aggregated into two categories: below 50% and those above 50%

### Data management and statistical analysis

The data were entered into Epi-Info software version 3.3.2. The STATA 12(STATA Corp, College Station, TX, USA) version was used for analysis. Descriptive analysis was undertaken. Multivariable logistic regression analysis with backward stepwise elimination was undertaken to determine factors independently associated with performance (good vs. poor). Potential predictors of CHW performance were selected from evidence and local knowledge and were retained where *p*<0.2 (21). The multivariable analysis shows AORs with corresponding 95% CIs.

The data were audio-recorded and transcribed for qualitative analysis. Thematic data analysis was used to develop themes from the transcripts. These were used to complement the findings from the quantitative analysis, especially in relation to factors associated with CHW performance. Quotations from the transcripts are included here to best illustrate the qualitative findings.

### Ethics approval

Ethics approval was granted by the Makerere University School of Public Health Higher degrees Research and Ethics Committee. Informed consent was sought from CHWs and caretakers of children participating in the study. Confidentiality of the study data was ensured by the principal investigator. All records were safely stored.

## Results

### Socio-demographic characteristics

Table 1 shows respondents’ socio-demographic characteristics. A total of 393 respondents participated in the study, a response rate of 92.8%. There was an almost equal distribution of females and males at 48 and 52%, respectively. All participants had attained at least a primary level of education although none had managed to go beyond the secondary level.

**Table 1:** Socio-demographic characteristics of respondents

Variable		Total *n*=393 (%)
Age	20–29	68 (17.3)
	30–39	168 (42.8)
	40–49	108 (27.5)
	50 >	49 (13.57)
Sex	Male	188 (47.8)
	Female	205 (52.2)
Education	Primary	157 (39.9)
	Secondary	236 (60.1)
Years of experience	1–2	48 (12.2)
	3–5	15 (3.82)
	Greater than 5	330 (84.0)
Marital status	Never married	59 (14.9)
	Married	328 (83.0)
	Separated/divorced	8 (2.0)
Another Source of income	Yes	134 (34.1)
	No	259 (65.9)

### Performance of CHWs

As shown in Table 2, the overall performance was low. There were 347 respondents (88.3%) with poor scores in managing malaria, diarrhea, and pneumonia, while 26 (6.6%) had moderate scores, and 20 (5.1%) had good scores. For knowledge assessment, 24 respondents (6.1%) had good scores, 121 (30.8%) had scored moderately, and 248 (63%) had poor scores. Knowledge on pneumonia was the lowest with 336 respondents (85%) scoring poorly and only 5 (1.3%) with a good score. Respondents had a better knowledge about malaria compared with diarrhea and pneumonia. There were 163 respondents (42%) with good scores and 50 (12.7%) with poor scores.

**Table 2:** Performance of CHWs in the management of malaria, diarrhea, and pneumonia

	Good score (75+)% *n* (%)	Moderate score (50–74)% *n* (%)	Poor score (0–49)% *n* (%)
Knowledge assessment *N*=393			
Malaria	163 (41.5)	180 (45.8)	50 (12.7)
Diarrhea	128 (32.6)	108 (27.5)	157 (39.9)
Pneumonia	5 (1.3)	52 (13.2)2	336 (85.5)
Total	24 (6.1)	121 (30.28)	248 (63.1)
Identification of signs, symptoms, and diagnosis in case scenarios *N*=393			
Malaria	125 (31.8)	161 (41.0)	107 (27.2)
Diarrhea	0 (0)	6 (1.5)	387 (98.5)
Pneumonia	0 (0)	4 (1.0)	389 (99.0)
Total	0 (0)	6 (1.5)	387 (98.5)
Prescription in case scenarios (right drug, dose, and instructions for mothers)			
Malaria, *N*=326	48 (14.7)	0 (0)	278 (85.3)
Diarrhea, *N*=58	0 (0)	1 (1.7)	57 (98.3)
Pneumonia, *N*=72	0 (0)	0 (0)	72 (100)
Total	5 (1.3)	10 (2.5)	378 (96.2)
Total performance	20 (5.1)	26 (6.6)	347 (88.3)

In the case scenarios, for the overall identification of signs, symptoms, and diagnosis, 387 (98.5%) respondents had a poor score and there were none with a good score. For both pneumonia and diarrhea, none of the CHWs had a good score, and for malaria, 125 (31.8%) had a score above 75% and 161 (41%) scored moderately.

Overall, for the prescription of drugs, 378 (96.2%) respondents had scored below 50%, and only 5 (1.3%) had scored above 75%. Malaria had relatively better scores with 48 (14.7%) attaining above 75%. For both diarrhea and pneumonia, no respondents had scores above 75%.

### Factors associated with performance of CHWs

The factors associated with the performance of CHWs were categorized into individual factors, social factors, and health system factors. Bivariate analysis was performed to determine the crude odds ratios (CORs).

**Table 3:** Summary of social demographic factors associated with performance of CHWs

	Performance categories		Crude analysis	
		
Variables	Score 0–49%, *n* (%)	Score 50–100%, *n* (%)	UOR (95% CI)	*p*
Age				
20–29	50 (16.7)	18 (19.2)	1	
30–39	124 (41.5)	44 (46.8)	0.99 (0.52–1.87)	0.965
40–49	87 (29.1)	21 (22.3)	0.67 (0.33–1.38)	0.276
50–59	38 (12.7)	11 (11.7)	0.80 (0.34–1.90)	0.619
Gender				
Male	137 (45.8)	52 (55.3)	1	
Female	162 (54.2)	42 (44.7)	0.68 (0.43–1.09)	0.109
Education level				
Primary	151 (40.9)	6 (25)	1	
Secondary	218 (59.1)	18 (75.0)	2.08 (0.81–5.36)	0.130
Marital status				
Never married	48 (16.1)	11 (11.7)	1	
Married/cohabiting	246 (82.3)	82 (87.2)	1.45 (0.72–2.93)	0.295
Separated/divorced	5 (1.7)	1 (1.1)	0.87 (0.09–8.24)	0.905
Another income source				
Yes	223 (74.6)	75 (79.8)	1	
No	76 (25.4)	19 (20.2)	0.74 (0.42–1.31)	0.305
Leadership position in community				
Yes	253 (84.6)	82 (87.2)	1	
No	46 (15.4)	12 (12.8)	0.80 (0.04–1.59)	0.533

OR=odds ratio. 95% CI=95% confidence interval.

### CHW management factors associated with performance

As shown in Table 3, none of socio-demographic factors were significantly associated with the performance of CHWs. As shown in Table 4, CHWs serving 100–200 households were less likely to have scores above 50% compared with those who were serving less than 100 households (COR 0.18; 95% CI 0.10–0.33). Similarly, CHWs serving more than 200 households were less likely to have scores above 50% compared with those who were serving 100 or less households (COR 0.24; 95% CI 0.13–0.46). Additionally, CHWs whose initial training lasted 2–3 days were more likely to have scores above 50% compared with those whose training lasted more than 3 days (COR 0.31; 95% CI 0.12–0.80). The CHWs managing two extra diseases were more likely to score above 50% compared with those who managed no extra illnesses (COR 4.63; 95% CI 2.17–9.88) and CHWs who met their supervisor once in the previous month were more likely to score above 50% compared with those who did not (COR 3.97; 95% CI 2.02–7.77).

**Table 4:** Summary of CHW management related factors associated with performance of CHWs

	Performance categories		Crude analysis	
		
Variables	Score 0–49%, *n* (%)	Score 50–100%, *n* (%)	UOR (95% CI)	*p*
Number of households served				
Less than 100	88 (29.4)	63 (67.0)	1	
100–200	125 (41.8)	16 (17.0)	0.18 (0.10–0.33)	0.001*
>200	86 (28.8)	15 (16.0)	0.24 (0.13–0.46)	0.001*
Duration of initial training				
2–3 days	253 (84.6)	89 (94.7)	1	
4–5 days	46 (15.4)	5 (5.3)	0.31 (0.12–0.80)	0.016*
Additional diseases managed by CHW in the community				
0	92 (30.8)	12 (12.8)	1	
1	159 (53.2)	53 (56.4)	2.56 (1.30–5.03)	0.007*
2>	48 (16.1)	29 (30.9)	4.63 (2.17–9.88)	0.001*
Financial incentives in the last month				
No	206 (68.9)	80 (85.1)	1	
Yes	93 (31.1)	14 (14.9)	2.58 (1.39–4.79)	0.003*
Meeting with supervisor in the last month				
No	196 (65.5)	11 (11.7)	1	
Yes	103 (34.5)	83 (88.3)	3.97 (2.02–7.77)	0.001*

* Denotes variables with significant *p*-values OR=odds ratio. 95% CI=95% confidence interval.

### Factors associated with CHW performance in the multivariable analysis

As shown in Table 5, the results show the multivariable analysis of independent factors predicting scores of above 50% performance among CHWs. The CHWs who had attained secondary education were more likely to score above 50% (AOR 2.72; 95% CI 1.50–4.92). The CHWs whose initial training lasted 2–3 days were more likely to perform better than those whose training lasted 3–5 days (AOR 0.1; 95% CI 0.04–0.41). The CHWs who served 100–200 households were less likely to score above 50% (AOR 0.24 95% CI 0.12–0.50) compared with those who served 100 or less. Similarly, the CHWs who serviced more than 200 households were less likely to score above 50% (AOR 0.22 95% CI 0.10–0.48). The CHWs who met with their supervisors the previous month were more likely to score above 50% compared with those who did not meet with their supervisors (AOR 2.52 95% CI 1.12–5.70).

**Table 5:** Factors that are independently associated with performance of CHWs

	Adjusted odds ratio (95% CI)	*p*
Education level		
Primary	1	
Secondary	2.72 (1.50–4.92)	0.001*
Duration of training		
2–3 days	1	
3–5 days	0.13 (0.04–0.41)	0.0001*
Number of households served		
Less than 100	1	
100–200	0.24 (0.12–0.50)	0.001*
>200	0.22 (0.10–0.48)	0.001*
Meeting with supervisor in last month		
Yes	1	
No	2.52 (1.12–5.70)	0.026*

* Significant *p*-value. OR=odds ratio. 95% CI=95% confidence interval.

A set of KIIs were conducted to supplement the quantitative findings. The results obtained from the qualitative analysis further supported the quantitative findings as indicated below. It was found that three main factors were major determinants of CHWs’ performance – training, incentives, and workload.

Regular training was reported as being useful in achieving good performance of CHWs. During discussions with the KIs, it was noted that in Lira, the CHWs for a long period of time had not received any refresher training in managing these illnesses. This resulted in many of them forgetting the key skills and knowledge needed for carrying out their duties. This is further illustrated by the following quotations:

We last had refresher training in 2011 … refresher trainings used to keep us knowledgeable on how to manage these childhood illnesses. (KII with CHW leader)

One of the supervisors goes on to state that

it has been long since they last had a refresher training, so they don't remember many of those concepts that were taught along ago … these are not trained medics so they should be continuously reminded using refresher trainings otherwise they loss the knowledge. (KII with member of DHOs’ office)

Similarly, in-kind community incentives were found to be important in keeping CHWs motivated. It was revealed that CHWs get incentives from communities that they serve, and this helped to keep them motivated. The community provision of various forms of incentives to their CHWs has resulted in reduced demand for financial incentives from the DHO. Both the CHWs and their supervisors perceive this as a good practice as illustrated in their voices below:

many of us get incentives from the community … they can give you a cup milk, or flour so even if you don't get official incentives as long as you work well you are appreciated. (KII with CHW coordinator)

They get incentives from the people they serve, it can be a cup of milk or ground nuts … people who get cared for thank them and this is encouraging to them. (KII with a member of DHO office)

Finally, the amount of work a CHW was supposed to undertake was identified as a hindrance to their performance. There was concern expressed that often CHWs get involved in many activities and that this can lead to poor performance. There is no structure in place that limits activities in which CHWs can indulge. Many CHWs were reported to be involved in managing many other illnesses. In addition, some were seen to be taking up political positions in the communities, which strained them and, therefore, negatively impacted on their performance as illustrated below.

… some CHWs are involved in many activities last week I called for a meeting one CHW was the chairman of the village, the mobilizer for TB programs and aspiring LC3 … they even are becoming politicians; they get overwhelmed, there should be a balance not too much but also not too little …. (KII with supervisor of CHWs)

## Discussion

The performance of CHWs in Uganda is lacking particularly for the management of pneumonia. The attainment of secondary-level education as well as having recently met with the supervisor was seen as ways of improving the performance of CHWs. Serving more than 100 households and a training duration of more than 3 days were factors that were negatively associated with performance. The qualitative findings showed a need for regular refresher training, in-kind incentives from community members, and a moderation in CHWs’ workload.

The level of performance of CHWs was lower than that reported in previous studies conducted in other areas of Uganda (11, 22). This could be because many of the previous studies were undertaken as part of a pilot project work. Project implementation is often well financed as there are typically project staff who can focus on the project and also dedicate funds for project activities. However, unlike project work, in the mainstream health system, these activities have to compete for time and resources with other activities. This can lead to reduced performance in the mainstream health system. This can even be more difficult in resource-constrained health systems like in Uganda where financing is limited (23).

Recent evidence suggests that pneumonia is more difficult to manage compared with malaria and diarrhea (11, 18). Further research indicates that performance of CHWs in the management of pneumonia is even worse when managed together with other illnesses (24). As mentioned by Kalyango et al. (11), it is important that training for CHWs is made a priority for improving knowledge in pneumonia case management. There is now sufficient evidence to show that when CHWs are well trained, they can sufficiently manage pneumonia (10).

At the time of this study, CHWs had spent 5 years without any form of refresher training. This finding may to a great extent explain the overall poor performance of CHWs because they need regular training in order to maintain a good level of knowledge and skills (25–28). It is therefore important to hold regular refreshment training for CHWs to ensure good job performance.

Furthermore, those CHWs whose initial training lasted 2 to 3 days were more likely to perform better than the rest of the CHWs who trained for more than 3 days. Previous studies show that CHWs benefit from 3-day training rather than longer periods (29). During the initial training, CHWs were introduced to basic concepts in relation to managing diseases but longer training may lead to information overload and thus result in less retention. There are many training sessions that last for months; however, it is important to note that they are mainly conducted on more educated CHWs unlike the CHWs in northern Uganda. It is, therefore, important to appreciate that even short training sessions covering 3 days can be effective as initial training for CHWs. Besides, after initial training many, CHWs are still lacking in knowledge and need to have their knowledge supplemented with regular refresher training (23, 30).

The CHWs who had met with their supervisors in the last 3 months were likely to perform better than those who had not. Studies have shown that support supervision is critical for improving the performance of CHWs (11). However, despite its importance, the implementation of support supervision has been identified as a weak link in many CHW programs (31, 32). Yet, it is not clear how this can be effectively implemented (33).

The CHWs whose catchment had less than 100 households performed better than those who covered more. Likewise, in the qualitative analysis, the workload was mentioned as a key determinant of performance. It has been recommended that CHWs are apportioned only a reasonable amount of work (34). When involved in so many programs, CHWs tend to get overwhelmed and perform poorly (2, 34–37). In Uganda, many program activities involve CHWs. The challenge, therefore, is to moderate these activities to reduce CHW workload.

### Methodological considerations

In case scenarios, each CHW managed a different child since the process of identification of signs and symptoms was the same for each child. However, prescriptions differed from one child to another because the diagnoses were different. Accordingly, some CHWs had to do more tasks when prescribing than others, and this created a level of non-uniformity in scoring. In order to address this, data about prescriptions were used only in describing performance. To determine the predictors of performance, we included only the identification of signs and symptoms. In the case scenarios, which were undertaken by all CHWs, the information was combined with knowledge assessment. The limitation of not capturing prescription ability was offset by the knowledge assessment questions that assessed the ability of CHWs to give proper treatment and medicine doses.

## Conclusions

The performance of CHWs is still inadequate. Pneumonia management had lowest scores and needs to be prioritized in the future training of CHWs. A pre-qualification test for CHWs may be helpful in ensuring that those who are the most competent are appointed. Support supervision and the recruiting of CHWs with at least secondary-level education may be helpful in improving the performance of CHWs. Health system managers need to ensure that the CHWs’ workload is moderated, and one key strategy is to ensure that they serve less than 100 households. Short training programs are beneficial but not sufficient and should be followed up with regular refresher training.

## Paper context

For over 20 years, a civil rebellion ravaged the Lira district. The recovery is ongoing, particularly in the health sector. The challenges of high child mortality burden, low human resources, and poor health-seeking behaviors persist. Use of CHWs to increase the proportion of children accessing care is a feasible solution to reduce some of these deaths. This article explores the performance of CHWs in managing childhood diseases and suggests some realistic solutions for addressing the gaps.
